# Identification of New Hepatic Metabolites of Miconazole by Biological and Electrochemical Methods Using Ultra-High-Performance Liquid Chromatography Combined with High-Resolution Mass Spectrometry

**DOI:** 10.3390/molecules29092160

**Published:** 2024-05-06

**Authors:** Michał Wroński, Jakub Trawiński, Robert Skibiński

**Affiliations:** Department of Medicinal Chemistry, Faculty of Pharmacy, Medical University of Lublin, Jaczewskiego 4, 20-090 Lublin, Poland; 54270@student.umlub.pl (M.W.); jakub.trawinski@umlub.pl (J.T.)

**Keywords:** Q-TOF, LC-MS, HLM, azole antifungal, SPE, metabolism, principal component analysis (PCA)

## Abstract

The main objective of this study was to investigate the metabolism of miconazole, an azole antifungal drug. Miconazole was subjected to incubation with human liver microsomes (HLM) to mimic phase I metabolism reactions for the first time. Employing a combination of an HLM assay and UHPLC-HRMS analysis enabled the identification of seven metabolites of miconazole, undescribed so far. Throughout the incubation with HLM, miconazole underwent biotransformation reactions including hydroxylation of the benzene ring and oxidation of the imidazole moiety, along with its subsequent degradation. Additionally, based on the obtained results, screen-printed electrodes (SPEs) were optimized to simulate the same biotransformation reactions, by the use of a simple, fast, and cheap electrochemical method. The potential toxicity of the identified metabolites was assessed using various in silico models.

## 1. Introduction

Miconazole, an azole antifungal drug, stands out for its broad-spectrum antimicrobial properties, effectively combating various pathogens responsible for dermatomycoses [1]. However, despite its efficacy in combating fungal infections, concern arises regarding the widespread use of miconazole and its potential impact on both human health and non-target organisms. This is particularly pertinent as miconazole has been detected in various environmental samples, raising concerns about its presence and potential impacts [2]. The dissemination of antifungal medications into the environment may potentially contribute to the spread of antimicrobial resistance [3]. Moreover, prolonged exposure to pollutants can also lead to their bioaccumulation in organisms. Similarly, miconazole’s pervasive presence in the environment is evidenced by its detection in living organisms, highlighting the risk it poses to biological metabolism. For instance, in a study conducted in Thailand, miconazole was detected in 17% of the samples of *Oreochromis niloticus* collected from the Chao Phraya River [4]. In another study, miconazole was detected in the plasma of eight different fish species collected from the Yangtze River with a maximum concentration of 0.00481 ng/L [5]. Additionally, in a study examining the liver tissue of twelve fish species collected from both the Yangtze River and the Pearl River, miconazole concentrations reached up to 432 ng/g [6]. Moreover, wild mussels (*Lasmigona costata*) collected from the far-downstream area of the Grand River in Canada showed miconazole concentrations with a maximum observed value of 1.22 ng/g. In caged mussels located near the downstream area, miconazole concentrations reached up to 1.71 ng/g [7].

Although miconazole is generally not administered orally, it is crucial to recognize that even in external applications, where absorption is limited, there remains a significant possibility of partial absorption. Additionally, it is substantial to highlight the fundamental concept that biotransformation processes may occur within the environment subsequent to the uptake of the chemicals by living organisms. While other tissues, such as the kidneys, intestines, lungs, brain, nasal epithelium, and skin, play a role in biotransformation, the liver, with its abundance of cytochrome P450 enzymes, predominantly orchestrates the majority of metabolic reactions in organisms [8]. Human Liver Microsomes (HLMs) are often employed as a simplified in vitro model system to study hepatic metabolic processes under controlled laboratory conditions. When supplemented with cofactors such as NADPH, HLMs provide a reliable evaluation of phase I hepatic metabolism. However, this approach has its limitations. While they contain cytochrome P450s, UGTs (UDP-glucuronosyltransferases), and flavin-containing monooxygenases (FMOs) in high amounts, the lack of enzymes such as N-acetyltransferases (NAT), glutathione-S-transferases (GST), and sulfotransferases (SULT) may prevent the production of certain metabolites present in vivo. Biological interferences such as the cellular matrix, phospholipids, and proteins complicate metabolite isolation, while highly reactive phase I metabolites may rapidly degrade or irreversibly bind to cellular macromolecules, hampering their detection in complex biological matrices. Moreover, the efficiency of reactions decreases over time, restricting the method’s utility as a straightforward source of metabolites. Consequently, researchers are actively exploring alternative methods for studying metabolism.

Understanding drug metabolism is crucial not only in pharmacological research but also because it directly impacts the pharmacokinetic behavior, efficacy, and potential toxicity of treatments. The metabolites formed from miconazole may possess their own activity, and their presence in the natural environment can have implications for various organisms. Miconazole, as a representative of azole antifungal drugs, exerts its antifungal activity by inhibiting cytochrome P450-dependent 14α-sterol demethylase. This inhibition disrupts ergosterol biosynthesis, ultimately compromising the integrity and function of the fungal cell membrane. Moreover, the antifungal mechanism of miconazole involves the generation of drug-induced reactive oxygen species (ROS) within the fungal organism, resulting in oxidative damage and ultimately culminating in cell death [9].

Nonetheless, it is possible that azole antifungal drugs could elicit biological effects in living organisms that extend beyond their intended therapeutic targets. They can interfere with various CYP enzymes [10,11] and 14-demethylase-dependent sterol synthesis pathways, which are prevalent not only in fungi but also in a wide range of organisms in nature [12,13]. The inhibition of sterol synthesis in animals and plants might have biological consequences, as sterols can serve as precursors for steroid hormones and are fundamental components of plasma membranes. Moreover, miconazole-induced ROS generation in HaCaT cells, an immortalized human keratinocyte line, resulted in oxidative stress and cell death [14].

Given the similarities between electrochemical and biological reactions, it is reasonable to assume that the redox mechanisms occurring at the electrode and within the human body share similar underlying principles [15]. The utilization of electrochemical methods allows us to observe which products coincide with those obtained using HLM and to identify substances that may arise in the environment through alternative mechanisms, particularly those involving redox reactions. This approach enables a more comprehensive understanding of potential chemical transformations that the substance may undergo, as well as facilitates the identification of differences between results obtained from HLM and electrochemistry. Electrode materials can effectively facilitate the oxidation or reduction of particular compounds, thus paralleling the role of enzymes involved in drug metabolism, allowing for controlled and precise investigations into how drugs are transformed within the body. Screen-printed electrodes (SPEs) are planar devices comprising plastic substrates coated with layers of electroconductive and insulating inks of controlled thickness. The introduction of this technology has facilitated the mass production of inexpensive disposable electrodes for conducting electrochemical experiments [16].

It should be noticed that electrochemical methods are also often utilized in environmental degradation research, including research on antifungal drugs. For instance, the E-peroxone process, utilizing a Pt anode and carbon-PTFE cathode at 15 °C, demonstrated superior efficacy in miconazole elimination, achieving faster results compared to conventional ozonation [17]. De Souza et al. also concluded that miconazole under optimal conditions exhibits high degradability, with 98% removal achieved within 5 min of ozonation and 94% removal achieved after 180 min of electrochemical oxidation on a boron-doped diamond anode with an applied current of 10 mA [18]. The study identified eight transformation products resulting from electrochemical oxidation and twelve products arising from ozonation. In another study by Ali et al., it was found that the combination of photodegradation under UV 254 nm light and electrochemical oxidation using a boron-doped diamond anode and an applied current of 10 mA successfully eliminated miconazole within a span of 45 min [19]. During these experiments, researchers identified eight products of anode oxidation, four products of UV photolysis, and eleven products formed in electrophotolysis.

In this study, we investigated the hepatic metabolism of miconazole established by utilizing an HLM assay in combination with UHPLC-HRMS. Through the acquired mass spectra, we successfully identified the metabolites of the drug, proposed a comprehensive metabolic pathway, and estimated the toxicity of the biotransformation products using various in silico models. Subsequently, building upon these findings, we optimized electrochemical parameters to simulate biotransformation reactions using SPEs. The utilization of multivariate chemometric analyses (Principal Component Analysis, PCA), facilitated the selection of optimal experimental conditions for the electrochemical experiments.

## 2. Results

### 2.1. Optimization of the LC-MS Method and Electrochemical Experiments

The chromatographic conditions were optimized to ensure a relatively short analysis time and to achieve good separation of the obtained products. The eluting solvents employed included an aqueous solution of formic acid (0.1%) and acetonitrile. The separation process was achieved by means of a gradient profile, as outlined in the Supplementary Material (Table S1 and Figure S1).

During the preliminary investigation of miconazole behavior on SPEs, different electrode materials and electrochemical experiments were examined. Electrochemical experiments were conducted using an Au SPE electrode, and a solution containing acetonitrile (due to miconazole’s limited solubility in aqueous solutions) with formate buffer in a 95:5 (*v*/*v*) ratio was selected as the optimal solvent composition. To determine the suitable pH of the buffer, cyclic voltammetry (CV) experiments (four cycles in the range from −2.1 V to 2.1 V with a scanning speed of 0.025 V/s) were conducted with buffer at pH 3, 7, and 9, and subsequent chemometric analysis guided the selection of the most suitable pH for the experiment. Cyclic voltammograms (presented in Figure S2) conducted at different pH values do not differ significantly, except for the cyclic voltammogram at pH 3. However, further chemometric analysis allowed for the determination of the optimal pH for conducting additional experiments. The conducted CV experiments allowed for a preliminary assessment of miconazole behavior under the investigated conditions. The selection of optimal CV parameters subsequently enabled the execution of chronoamperometric analyses over a wider range of potentials. Finally, the electrochemical behavior of miconazole in the buffer with pH 3 was evaluated by conducting chronoamperometry experiments using specific potentials: 0.8, 1.2, 1.6, 2.0, and 2.4 V, with each experiment lasting for 15 min, and with an interval time of 0.5 s.

### 2.2. Metabolite Identification and Transformation Pathway

**Figure 1:**
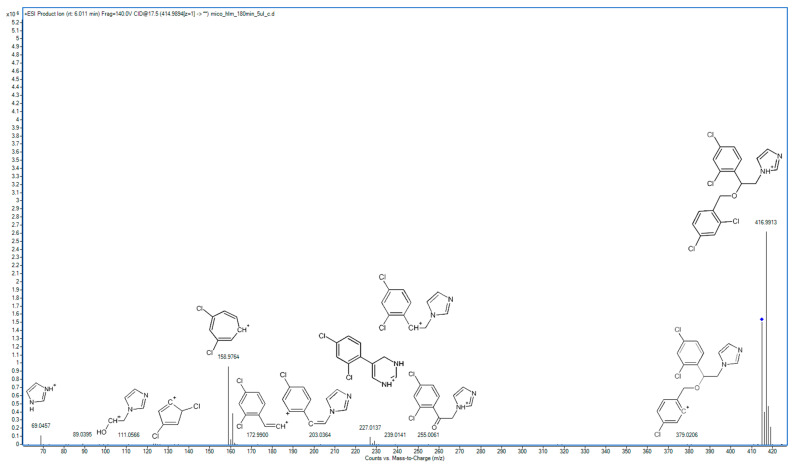
MS/MS spectrum and fragmentation pattern of miconazole.

**Figure 2:**
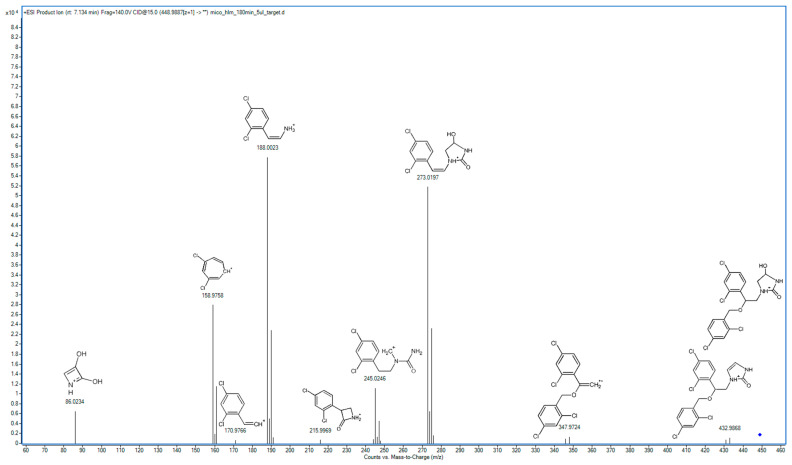
MS/MS spectrum and fragmentation pattern of M1.

**Figure 3:**
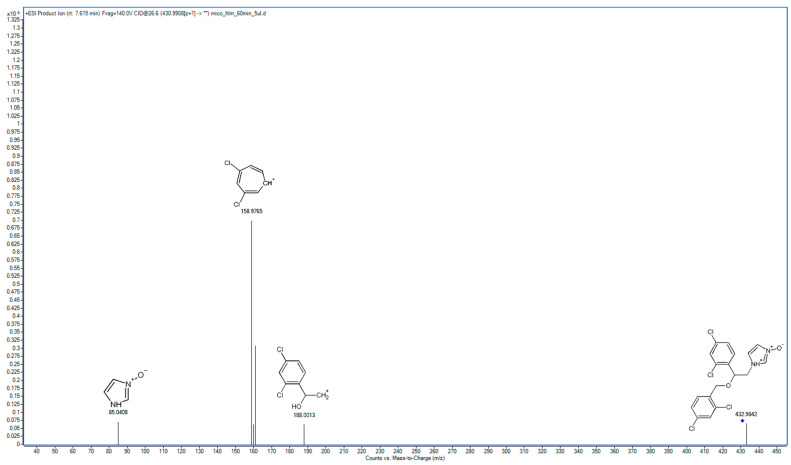
MS/MS spectrum and fragmentation pattern of M2.

**Figure 4:**
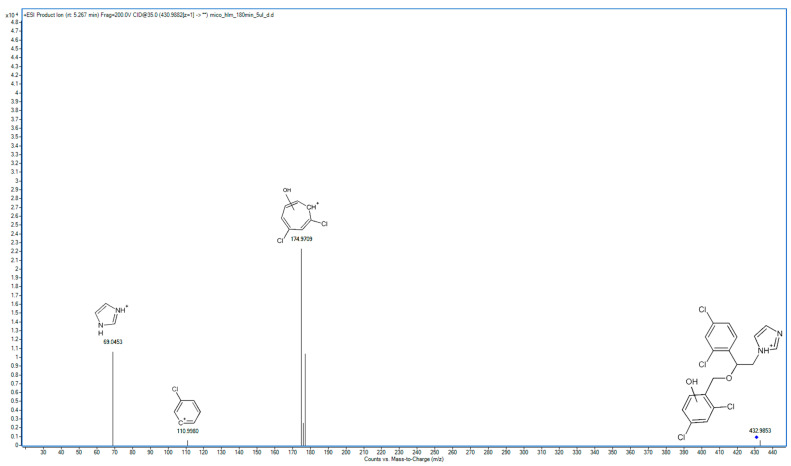
MS/MS spectrum and fragmentation pattern of M3.

**Figure 5:**
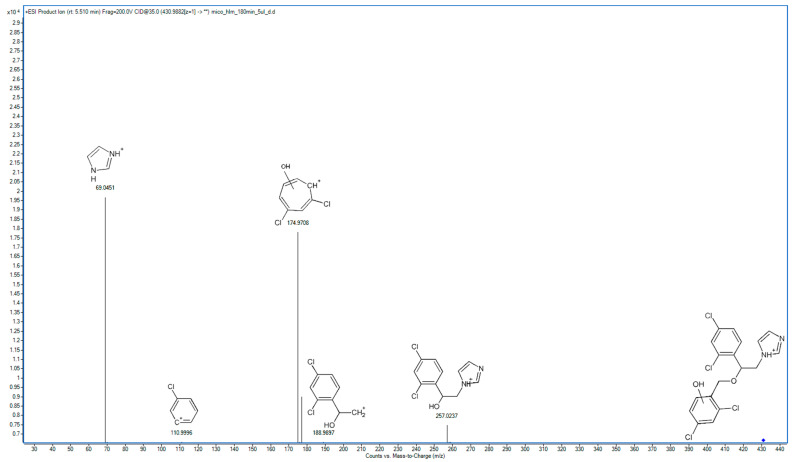
MS/MS spectrum and fragmentation pattern of M4.

**Figure 6:**
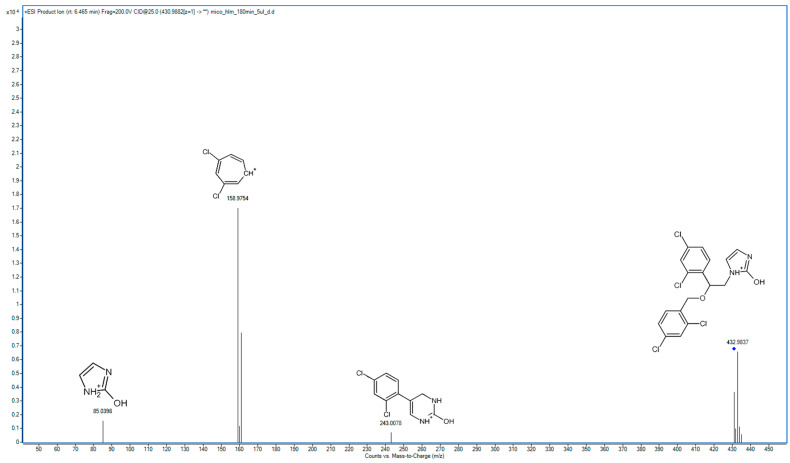
MS/MS spectrum and fragmentation pattern of M5.

**Figure 7:**
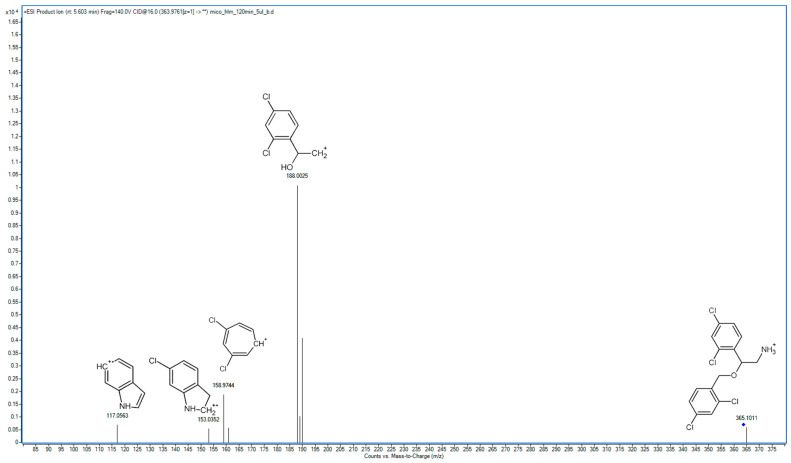
MS/MS spectrum and fragmentation pattern of M6.

**Figure 8:**
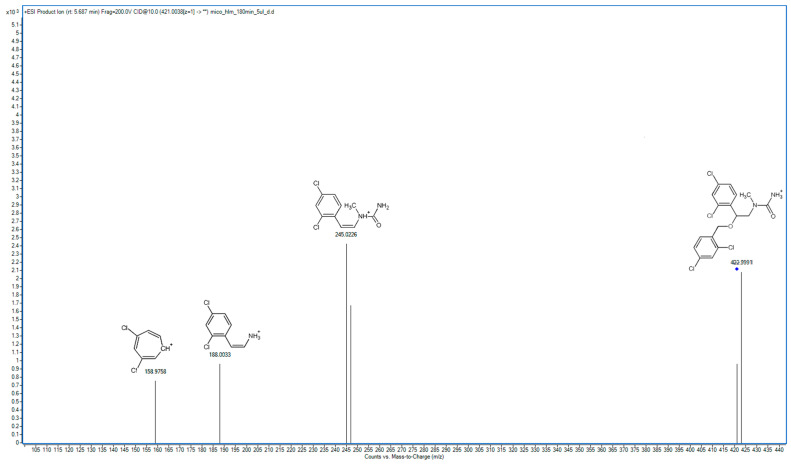
MS/MS spectrum and fragmentation pattern of M7.

In the following study, seven hepatic metabolites of miconazole were found and identified using high-resolution mass spectrometry. The fragmentation patterns of miconazole and its metabolites are summarized in Table 1, while the fragmentation MS/MS spectra are presented in Figure 1, Figure 2, Figure 3, Figure 4, Figure 5, Figure 6, Figure 7 and Figure 8.

The spectrum of miconazole, presented in Figure 1, is characterized by prominent fragments originating from the detachment of the dichlorobenzyl moiety, particularly at the oxygen linkage site. The most conspicuous peak in the spectrum originates from the detached fragment (158.9764 *m*/*z*). The remaining structure is represented by a fragment at 255.0061 *m*/*z*. The fragmentation process of this fragment involves the successive loss of oxygen (resulting in a fragment at 239.0141 *m*/*z*), which in turn decomposes into a fragment without a chlorine atom (203.0364 *m*/*z*) or, alternatively, undergoes fragmentation on the opposite side, leading to the removal of a carbon atom from the imidazole ring and subsequent rearrangement of the structure, as evidenced by the fragment at 227.0137 *m*/*z*. In some instances, the entire imidazole ring may be lost, leading to the formation of a fragment at 172.9900 *m*/*z*. The spectrum also contains visible fragments indicating the preservation of imidazole (69.0457 *m*/*z*) and 2-(1-imidazolyl)ethanol (111.0566 *m*/*z*). Another noteworthy fragment in the spectrum is observed at 379.0206 *m*/*z*, which signifies the detachment of a chlorine atom.

M1 represents the derivative where hydroxylation and oxygenation occurred at the carbon atoms within the imidazole ring (Figure 2). The primary peak in the M1 spectrum corresponds to 2-(2,4-dichlorophenyl)ethylamine (188.0034 *m*/*z*), and the main peak from the parent spectrum (158.9760 *m*/*z*) is also evident in the M1 spectrum. Notably, the spectrum lacks the fragment observed in the parent spectrum fragment resulting from the detachment of dichlorobenzyl. However, one of the main peaks is represented by a fragment with an *m*/*z* value of 273.0218, which indicates the presence of two hydroxyl groups and two hydrogen atoms within this fragment. The detachment of oxygen and carbon from the structure at 273.0218 *m*/*z* results in the formation of the ion at 245.0246 *m*/*z*. The fragment originated from the detachment of the imidazole moiety, with an ion at *m*/*z* 170.9801, indicating that redox reactions occurred outside of the phenyl ring. Additionally, the fragment with an *m*/*z* value of 86.0242 represents dihydroxyazetidine, providing direct evidence of a double oxidation within the imidazole structure. The fragment at 430.9880 *m*/*z* resulting from dehydrogenation indicates that *N*-oxidation within the imidazole moiety did not occur. While metabolic reactions that occur in the case of M1 are not frequent, there are documented cases in which CYP enzymes are responsible for such reactions. For instance, a comparable metabolite in humans involving an imidazole moiety has nafimidone [20]. The double oxidation of the monosubstituted imidazole ring has been documented in the metabolism of econazole [21], which is another representative imidazole antifungal. Based on these, we assume that oxygenation took place at the C2 position and hydroxylation occurred at the C4 position of the imidazole ring.

M2 has been identified as a product of *N*-oxidation, primarily based on the presence of a distinct fragment in its spectrum with an *m*/*z* value of 85.0408, indicating oxidation within the imidazole moiety (Figure 3). Notably, the primary peak in the spectrum corresponds to the detachment of the dichlorophenyl moiety (158.9765 *m*/*z*), suggesting that oxidation did not occur within this moiety. Additionally, the spectrum reveals an ion peak from 2-(2,4-dichlorophenyl)ethylamine (188.0013 *m*/*z*), indicating that similar processes occurred in these compounds. The nitrogen atom’s basicity being blocked resulted in a significant extension of the retention time in the applied chromatographic system. Notably, the MS/MS spectrum of M2 is notably deficient, a characteristic often observed in transient *N*-oxides.

M3 and M4 are isomers of metabolites in which hydroxylation has occurred within the phenyl ring. In the spectrum of M3, the main ion is observed at *m*/*z* 174.9709, replacing the fragment at 158.9764 *m*/*z* from the parent compound’s MS/MS spectrum (Figure 4). This indicates that hydroxylation took place in the dichlorobenzyl fragment. A prominent peak with an *m*/*z* value of 69.0453 represents imidazole. Additionally, the spectrum features a visible fragment at *m*/*z* value 110.9980, representing chlorophenyl.

In the spectrum of M4 (Figure 5), two primary peaks are notable: one at 69.0451 *m*/*z* and the other at 174.9708 *m*/*z*. These peaks correspond to the imidazole and hydroxylated dichlorobenzyl fragments, respectively. The peak at 257.0237 *m*/*z* represents the remaining structure after the detachment of the 174.9708 *m*/*z* fragment, indicating a structure in which oxidation took place. Further fragmentation of this fragment leads to the appearance of a peak at 188.9897 *m*/*z*, which occurs following the detachment of the imidazole moiety. The spectrum also includes the fragment at 110.9980 *m*/*z*, as observed in the M3 spectrum, indicating that hydroxylation of the phenyl ring results in different fragmentation behavior, making it more prone to the loss of a chlorine atom during fragmentation. Drawing from the insights provided by Rietjens et al., who presented a hypothesis for predicting the regioselectivity of the cytochrome P450 hydroxylation of halogenated benzenes, we can infer that in the case of M3, hydroxylation occurred at the C4 position of the benzene ring, while in the case of M4, hydroxylation took place in the meta position relative to the chloro substituents [22]. De Souza et al. have previously identified a product in which hydroxylation took place in the benzene ring of miconazole [18]. However, this was not a miconazole metabolism product, but a transformation product formed during the ozonation and electro-oxidation of miconazole.

M5 represents another mono-oxygenated metabolite, with hydroxylation occurring within the imidazole moiety. Consistent with the spectra of other metabolites, a peak with an *m*/*z* value of 158.9754 was the most prominent in its spectrum (Figure 6). The presence of a peak at 85.0398, which replaces the fragment representing imidazole (69.0457 *m*/*z*), provides direct evidence that hydroxylation took place within the imidazole moiety. Additionally, the spectrum features a noticeable peak at 243.3078, indicating that the fragment visible in the parent spectrum at 227.0137 *m*/*z* underwent hydroxylation. Based on the metabolism of econazole, we assume that in the case of M6, hydroxylation takes place at the carbon atom positioned between the two nitrogen atoms within the imidazole moiety [21].

The last two metabolites, M7 and M6, were formed by the successive breakdown of the imidazole moiety of M1. Initially, M1 underwent a transformation resulting in the formation of M7. Subsequently, M7 underwent further degradation, leading to the complete degradation of the imidazole moiety and the formation of the free amine M6. The primary peak in the spectrum of M6 (Figure 7) corresponds to a fragment (188.0025 *m*/*z*) that results from the detachment of the dichlorobenzyl fragment and the loss of an amine group. The spectrum of M6 prominently features a peak originating from dichlorobenzyl (158.9744 *m*/*z*). Another fragmentation pathway involves the detachment of the dichlorobenzoxy moiety, as well as the loss of one chlorine atom from the left benzene ring, resulting in the rearrangement of a structure in a fragment with an *m*/*z* of 153.0352. Subsequently, the second chlorine atom is lost from the benzene ring, leading to the formation of a fragment at 117.0563 *m*/*z*. The rearrangement of the structure likely occurred due to the presence of the free amine group, which served as an electron donor to the carbon atom in the benzene ring after the detachment of the chlorine atom. The formation of this product in degradation studies was also reported by de Souza et al. [18].

**Figure 9:**
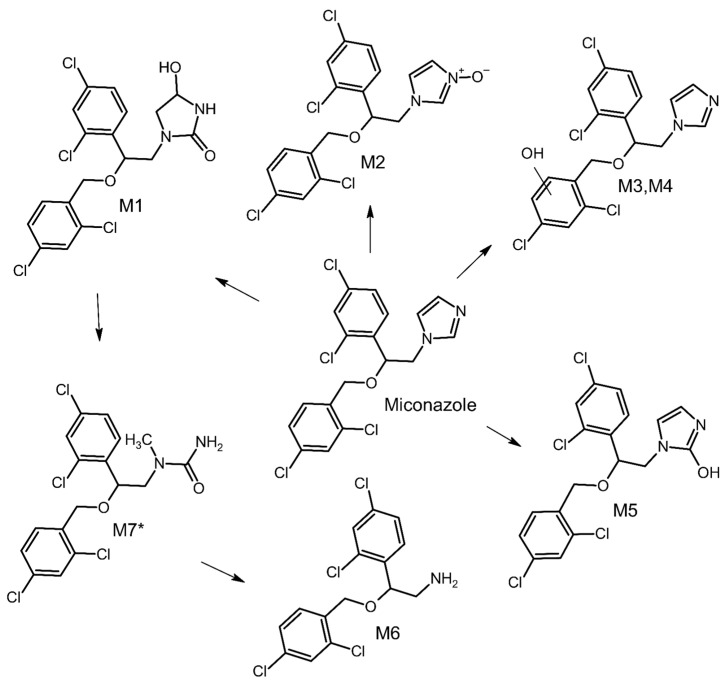
The hepatic metabolic pathway of miconazole (* Metabolite not formed by electrochemical experiments).

M7 was formed through the detachment of the hydroxyl group along with a carbon atom from the imidazole ring. In the spectrum of M7 (Figure 8), there is also a peak at an *m*/*z* value of 158.9758, which, as with other metabolites, excludes metabolic changes within the structure of dichlorobenzyl. The primary peak in the spectrum (245.0226 *m*/*z*) corresponds to the structure formed after the detachment at the above-mentioned 158.9758 *m*/*z*. The fragment at 245.0246 *m*/*z* was also visible in the MS/MS spectrum for M1, suggesting that M7 is formed following a partial breakdown of the imidazole moiety in the M1 structure. Additionally, there is a visible prominent fragment with an *m*/*z* of 188.0033, which corresponds to 2-(2,4-dichlorophenyl)ethylamine. The metabolic pathway of miconazole is presented in Figure 9.

### 2.3. Biotransformation of Miconazole

The kinetics of miconazole in in vitro biotransformation were assessed using an HLM assay, focusing on the abundance of the parent ion. The study encompassed a range of incubation times (0–180 min) and demonstrated a modest metabolism of the analyzed drug. After 120 min, the drug exhibited a metabolism rate of 8%, and then the reactions of biotransformation slowed down.

**Figure 10:**
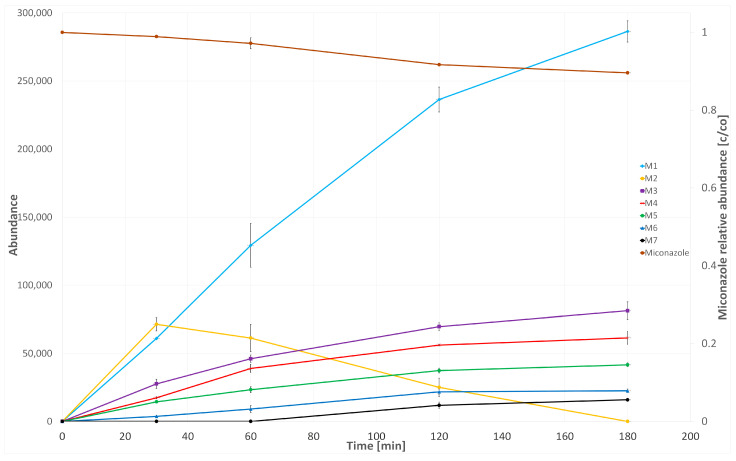
Formation of miconazole metabolites during HLM incubation.

M2 emerged as the primary metabolite following a 30 min incubation, but its formation rates markedly decreased thereafter. In contrast, the formation rates of all the other metabolites only begin to decelerate after 120 min of incubation. Taking this into account, to perform chemometric and qualitative analyses, the time of 120 min of HLM incubation with miconazole was selected. The evolution profiles of miconazole metabolites obtained during incubation with HLM are presented in Figure 10.

### 2.4. Multivariate Comparison of HLM Metabolites and Electrochemical Products

**Figure 11:**
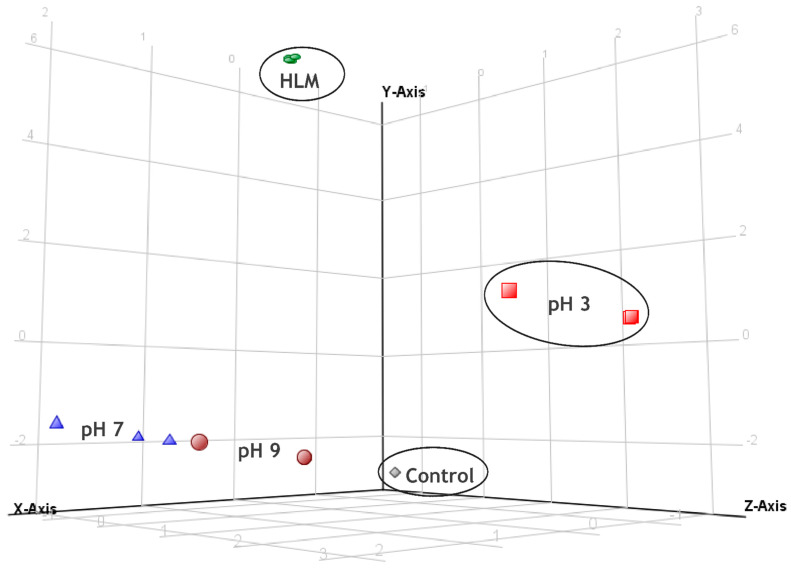
A 3D plot of principal component analysis comparing the biological HLM and electrochemical profiles of miconazole (Y-Axis—PC1, Z-Axis—PC2, X-Axis—PC3).

A multivariate chemometric analysis was carried out to assess qualitative and quantitative differences in the recorded profiles of miconazole metabolites from HLM and products resulting from electrochemical experiments. In this study, a total of twenty-five chromatograms (five replicates for five experiments) were collected in TOF (MS) mode and were aligned using MPP software, resulting in 415 distinct entities. Applying build-in MPP filtration techniques, including filtering by flags and sample abundance, followed by volcano plot (fold-change threshold on the level not less than 2.0, and *p* = 0.05), resulted in the selection of a final set of 17 entities for further chemometric analysis. Fifteen of them correspond to the ions of metabolites or their isotopic ions. An ion with an *m*/*z* of 259.0214 corresponds to the product of electrochemical degradation (observed in the pH 7 CV experiment). By contrast, an ion with an *m*/*z* of 397.0262 aligns with the retention time of M3, which loses a chlorine atom in the ion source of the mass spectrometry. The list of entities included in PCA is presented in Table S2. In the presented PCA (Figure 11), 93.43% of the total variance was explained by the first three principal components (PC1—69.39%, PC2—16,82%, and PC3—7.22%). A scree plot is presented in the Supplementary Material (Figure S4).

The distinct separation of control samples from the rest of the experimental samples serves as confirmation of metabolic reactions taking place. It is noteworthy that the PCA results revealed that the most accurate simulation of HLM was achieved with Au electrodes at pH 3. Samples at pH 3 were the closest to the HLM samples on the Y-axis, representing the highest variance. Conversely, experiments conducted at pH 7 or pH 9 showed greater separation from the HLM samples compared to the pH 3 samples. Moreover, the pH 3 samples were even more distant from the control samples than the samples from other electrochemical experiments. Further data analysis revealed that the most important ions responsible for the separation of pH 3 and HLM samples from the remaining experiments represented M2, M3, M4, and M6 metabolites (loadings corresponding to 4, 5, 8–11, 13, and 14 entities in Figure S5), which were formed in relatively large quantities in these two experiments. On the other hand, samples obtained in pH 7 and pH 9 were marked by low (or even zero) abundance of the statistically significant ions. Based on these results, further chronoamperometric electrochemical experiments were performed at a pH of 3.

### 2.5. Electrochemical Experiments

The primary electrochemical products observed during this study were M2 and M6 (Figure S3). The product formed from the decomposition of miconazole, M6, was readily formed in electrochemical reactions, and its formation demonstrated an increase as the applied potential was raised. Its peak formation potential occurs at 2.4 V and it was the sole metabolite formed in a substantial quantity under these conditions. M2, recognized as the *N*-oxide metabolite, was the most easily formed oxidation product and the main metabolite formed at low potentials, with its potential range of 0.8 to 1.2 V. Specifically, the peak formation for M2 was situated at a potential of 0.8 V, although its formation declined at higher potentials. Nonetheless, an optimal condition for its synthesis was observed at a potential of 2.0 V. In a manner similar to the *N*-oxide, in the case of M3, M4, and M5, two distinct potential optima were also observed. The first peak formation potential for M3 occurs at 0.8 V, while for M4 and M5, it was at 1.2 V. Nevertheless, a significantly greater amount of these metabolites was formed at the second optimum of 2.0 V. At this potential, M1 was also created with maximum effectiveness, even though it was formed only in small quantities at low potentials. However, as the potential increased further, the efficiency of their formation began to decrease, likely due to increased decomposition of the resulting electrochemical products. As a consequence of the inefficient formation of M1, the formation of M7 using electrochemical methods could not be obtained; however, its formation in the biological experiment was also very slight. It should be noted that electrochemical methods helped in the synthesis of minor metabolites, for example, M6, with greater efficiency, which was an advantage in terms of its identification and structural characterization. The high efficiency in the formation of M2 by electrochemical methods has assisted in the analysis of its MS/MS spectra.

These findings suggest the existence of a distinct reaction mechanism at potentials within the range of 0.8–1.2 V in contrast to those around 2.0 V. The anodic oxidation of organic compounds can take place through two distinct mechanisms: direct electron transfer (DET) and oxidation mediated by hydroxyl radicals. DET is a process where electrons are directly transferred between an electrode and a redox-active molecule or species in solution, occurring without the intervention of redox mediators. On the other hand, at high anodic potentials in the region of water discharge, organic compounds can also be oxidized due to the participation of intermediates of oxygen evolution.

### 2.6. Toxicity

In this study, the toxicity assessment was performed using the in silico approach. Although the in vivo methods (utilizing living organisms) are still considered the most reliable, they possess several serious drawbacks as they are expensive, time-consuming, and, above all, questionable from an ethical point of view. Therefore, it is considered important not to use them unless it is necessary. Thus, other approaches, such as methods based on computational modelling (in silico) have gained great popularity recently. Obviously, their reliability varies depending on the calculation method or the applicability domain, but it is widely accepted that if they are properly used, they can be successfully applied at the preliminary stage of research [23,24,25].

In order to maximize the reliability of the estimations, we applied more than one model for each toxicity category (unless only one model was available). In cases where a large number of models were used, the outcomes were submitted to PCA in order to facilitate interpretation of the results as well as to graphically present the relationships between the metabolites and applied QSAR models. It should be noted that scores of PCA represent the identified metabolites and the parent compound, while loadings represent the toxicity models.

#### 2.6.1. Aquatic Toxicity

The descriptions of twenty-three models used to calculate the toxicity of miconazole metabolites towards aquatic organisms are summarized in Section S1. Due to a high number of applied toxicity models, the obtained results were divided into three subgroups: *D. magna*, Fish (including zebrafish and *F. minnow*), and algae, and subsequently submitted to PCA (when interpreting the results of chemometric analysis, it should be remembered that toxicity decreases in parallel with increasing values such as LD_50_, LC_50_, EC_50_, etc.). All raw data concerning aquatic toxicity are presented in the Supplementary Material (Tables S3–S5).

##### Fish

**Figure 12:**
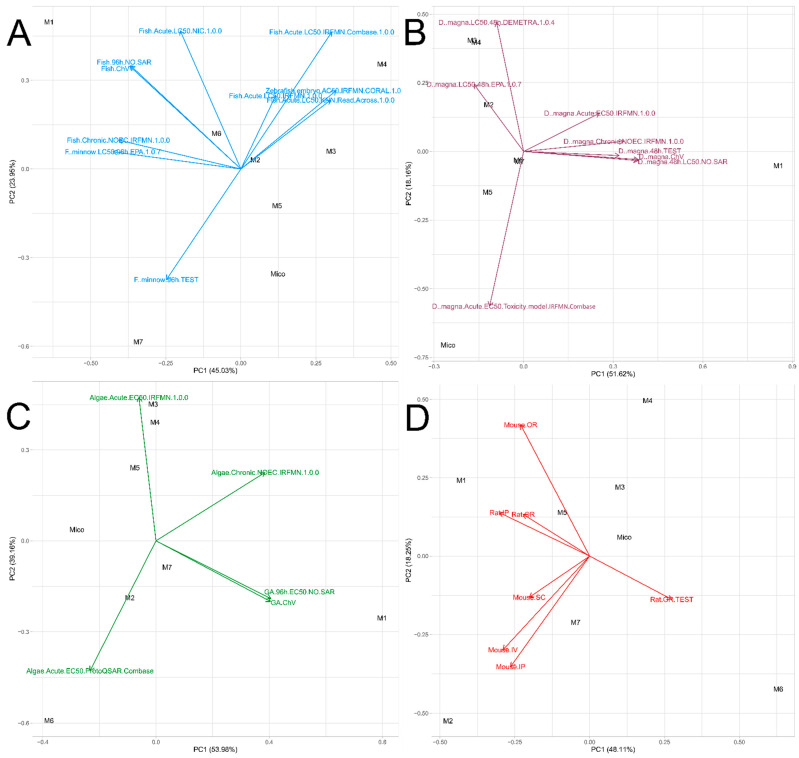
Plot of principal component analysis of calculated aquatic toxicity of miconazole metabolites towards different aquatic organisms ((**A**)—fish, (**B**)—*D. magna*, (**C**)—algae, (**D**)—rodents).

In order to facilitate the interpretation of the fish toxicity results predicted by 10 models, PCA was performed. As shown in Figure 12A, there are four pairs of highly correlated (giving similar results) variables. The first two pairs, Zebrafish Embryo AC_50_ IRFMN/Coral Fish Acute LC_50_ KNN/Read-Across and Fish Acute LC_50_ IRFMN–Fish Acute LC_50_ IRFMN/Combase, predicted the lowest toxicity for the products of aromatic hydroxylation (M4 and M3) and relatively low toxicity for the products of imidazole oxidation and its total decomposition (M2, M5, and M6). On the other hand, the parent compound and M7 were defined as generally the most toxic. Interestingly, the *F. minnow* 96 h T.E.S.T. model gave opposing results. The third pair of correlated variables consists of the ECOSAR predictions: Fish 96 h NO SAR–Fish ChV (predictions of Fish Acute LC_50_ NIC were quite close to this pair). Those models predicted the lowest toxicity for M1, moderate toxicity for M2 and M6, and the highest toxicity for M5 and miconazole. The results predicted by the last pair of correlated variables (*F. minnow* LC_50_ 96 h EPA–Fish Chronic NOEC IRFMN) were generally close to the above-mentioned lowest-toxicity estimations for M1 and highest estimations for M5 and the parent compound. Although it may be difficult to unambiguously indicate the most and the least harmful compounds due to the diversity of the applied models’ predictions, it can be assumed that the metabolites are generally less toxic than the parent ion, and hydroxylation of the imidazole ring (M5) changes its properties to the least extent. On the other hand, the toxicity of the major metabolite (M1) should be considered as relatively low.

##### *D.* *magna*

In contrast to fish, the majority of the models predicting toxicity towards daphnia gave highly correlated results (Figure 12B), indicating the lowest toxicity for M1 and generally the highest for miconazole. Predictions for the remaining metabolites were diverse; however, the outcomes were generally closer to the parent compound (higher toxicity) than to M1 (similarly as in the other aquatic toxicity categories and, in this case, the higher loading values correspond to decreased toxicity, which is justified by increasing LC_50_ or EC_50_ values). Interestingly, one pair of correlated models (*D. magna* LC_50_ 48 h DEMETRA–*D. magna* LC_50_ 48 h EPA) indicated that aromatic hydroxylation (M3 and M4), and, to a lesser extent, *N*-oxidation (M2) results in a significant decrease of the toxic properties. On the other hand, the most outlying model (*D. magna* Acute EC_50_ Toxicity model IRFMN/Combase) gave opposite results, also predicting miconazole as the least toxic.

##### Algae

The results obtained for algal toxicity were the most divergent (Figure 12C). Only two models (both provided by ECOSAR) gave similar results, predicting M1 as the least toxic, while miconazole, along with M5, was the most harmful. The lowest toxicity of all hydroxylated metabolites (M3–M5) was predicted by the Algae Acute EC_50_ IRFMN model (conversely, the opposite results were generated by the Algae Acute EC_50_ ProtoQSAR/Combase model, which predicted the lowest toxicity for M6).

#### 2.6.2. Acute Toxicity to Rodents

Toxicity to mice and rats was calculated using seven models: six provided by Percepta (rat and mouse oral, mouse intravenous, intraperitoneal and subcutaneous, and rat intraperitoneal) and one provided by T.E.S.T. (rat oral, nearest neighbor model with non-relaxed fragment constraint). All outcomes were expressed as LD_50_, using log_(mg/kg)_ values (raw data are presented in Table S6). As can be seen in the PCA plot (Figure 12D), there are two groups of correlated variables: the first one consisting of Mouse SC, IP, and IV models, and the second one grouping Rat IP, OR, and Mouse OR (a slightly more outlying vector) together. However, these two groups of variables are weakly correlated with each other, as the angle between them is close to 90°. On the other hand, the variable representing the T.E.S.T. model (Rat OR) is inversely correlated with the second group of variables (which means that this model predicted contrasting results). Considering the compounds, it can be seen that miconazole, M3, and M5 possess rather similar toxic properties. M1 is significantly less toxic according to the second group of variables (and more toxic according to the Rat OR T.E.S.T. model. The results should be interpreted in the same way as in the case of aquatic toxicity). Inverse results were obtained for M6. On the other hand, the first group of models predicted M2 as the least toxic and M4 as the most harmful compound. Based on the obtained results, it can be seen that there is no apparent dependence between the structure of the metabolite and its toxicity to rodents.

#### 2.6.3. Mutagenicity and Developmental Toxicity

The mutagenic potential (expressed as a probability of the positive outcome of the Ames test) of the miconazole metabolites was estimated using two models provided by Percepta and T.E.S.T. software. According to the Percepta model, none of the discussed compounds possess mutagenic potential. However, M1, M2, and M7 are marked with a slightly higher probability of such action. On the other hand, T.E.S.T. software predicted significantly higher values. According to this model, the parent compound possesses a borderline probability of mutagenic action (0.5 but defined as ‘mutagenic positive’ by the software). Moreover, the predicted mutagenicity of miconazole *N*-oxide (M2) was exceptionally high (0.82). The remaining metabolites were defined as less toxic than the parent compound.

Developmental toxicity was calculated using one model provided by the T.E.S.T. software. The obtained outcomes indicate that all the metabolites may possess greater toxic potential than miconazole itself (which is in fact the only compound defined as non-developmentally toxic), especially in the cases of M1 (the most abundant metabolite) and M7.

Raw data concerning mutagenicity and developmental toxicity are presented in Table S7 in the Supplementary Material.

#### 2.6.4. Receptor-Mediated Toxicity

The endocrine-disruption potential (receptor-mediated toxicity) was estimated using the Endocrine Disruptome platform. The following target receptors were chosen: androgen (AR), estrogen alpha (ERα) and beta (ERβ), progesterone (PR), glucocorticoid (GR), mineralocorticoid (MR), liver X alpha (LXRα), retinoid X alpha (RXRα), peroxisome proliferator-activated receptor gamma (PPARγ), thyroid alpha (TRα), and beta (TRβ). In the cases of AR, ERα, ERβ, and GR receptors, the antagonistic interactions were also studied.

The obtained results are shown in Table S8. Numerical values presented in the table correspond to the binding probability (increasing in parallel with decreasing numbers); however, they should be compared only column-wise, e.g., a value of −9.8 represents a high binding probability in the case of MR, but is still low in the case RXRα. Therefore, each receptor should be considered separately. Miconazole and the majority of its metabolites possess a high potential of binding to MR (two exceptions are M5 and M7, where moderate-high and moderate-low probabilities were observed, respectively). All the analyzed compounds can also bind to GR (moderate-low probability) and AR as the antagonists of this receptor (also moderate-low probability, except M7, where a moderate-high probability was predicted). Most of the compounds may also bind to TRβ, albeit at a moderate-low level of probability. Among the metabolites, M1, M2, and M5 can be viewed as the most harmful. M1 binds to four receptors at moderate-low levels (the same number as miconazole); however, it is the only compound possessing high probability in the case of two proteins, MR and ERβ (as its antagonist). M2 and M5 bind to six and seven receptors at moderate-low levels (both can strongly bind to MR). M7 can also be considered as more toxic than the parent compound (moderate-low binding to five receptors, moderate-high to one, and high to one).

Based on the obtained results, it can be seen that the majority of the metabolites possess higher endocrine-disrupting potential than the parent compound (only M4 and M6 have definitively lower binding probabilities). Noticeably, the most harmful compounds were formed as a result of imidazole ring hydroxylation/oxidation, and even partial decomposition of this moiety did not reduce such properties (M7). On the other hand, the total degradation of imidazole, resulting in the formation of primary amine, did not significantly alter the studied properties. In addition, hydroxylation of the dichlorophenyl ring did not increase the toxicity, and in the case of M4, a decrease was observed.

## 3. Materials and Methods

### 3.1. Chemicals and Reagents

Miconazole nitrate and sodium phosphate dibasic anhydrous salt were obtained from Sigma-Aldrich (St. Louis, MO, USA). LC-MS-grade acetonitrile and water, as well as LC-grade water, were purchased from Witko (Łódź, Poland). MS-grade ammonium formate was obtained from Agilent Technologies (Santa Clara, CA, USA) and MS-grade formic acid (98%) was acquired from Avantor Performance Materials Poland S.A. (Gliwice, Poland).

### 3.2. In Vitro Simulation of Metabolism with HLM

For biotransformation experiments, a stock solution of miconazole in acetonitrile was diluted to working concentrations (1 mM) with ultrapure water. An in vitro biotransformation assay was performed using HLM fractions. The incubation mixture consisted of 0.05 mM of the substrate, 50 mM phosphate buffer at pH 7.4, and 0.5 mg/mL HLM. Following a 2 min pre-incubation period at 37 °C, the metabolic reactions were initiated by the addition of 10 μL NADPH (20 mM) with gentle, constant shaking in an Eppendorf ThermoMixer C equipped with Eppendorf ThermoTop (Eppendorf AG, Hamburg, Germany), After 0, 30 60, 120, and 180 min of incubation, the reaction was terminated by the addition of 40 μL ice-cold acetonitrile–methanol mixture (1:1). The resulting samples were then centrifuged at 16,000 rpm for 10 min at 4 °C, and the supernatants (40 μL) were transferred into the vials for UHPLC-HRMS analysis in two replications. The same procedure was applied to the negative control samples, with the exception that no NADPH solution was added.

### 3.3. Electrochemical Studies

The constant potential amperometry experiments were conducted using the Autolab/PGSTAT302N modular potentiostat/galvanostat, an electrochemical measuring instrument manufactured by Metrohm Autolab (Utrecht, Netherlands). The experiments were controlled using Nova 2.1.5. software. The electrochemical behavior of miconazole was investigated using a gold (Au) SPE, specifically utilizing the 220BT electrode model. The electrode systems consist of a flat ceramic card with a circular gold working electrode (4 mm diameter with a 0.11 cm^2^ surface area), a gold auxiliary electrode, and a silver pseudo-reference electrode. Before conducting experiments, each electrode underwent a conditioning process. For this purpose, a 50 µL drop of a supporting electrolyte without substance was placed on the electrode and three cycles of cyclic voltammetry were performed in the potential range of −2.1 V to 2.1 V with a scanning speed of 0.1 V/s. A stock solution of miconazole (10 mM) was diluted in a supporting electrolyte to prepare a working solution (0.025 mM). Optimization experiments were conducted using the 20 mM formate buffer at pH values of 3, 7, and 9. All the experiments were conducted at room temperature, and a 50 µL drop of the solution was used to cover the SPE surface. The SPE was positioned horizontally and was placed for the duration of the experiment in a cell for SPE (Metrohm Autolab, Utrecht, Netherlands) to prevent evaporation of the droplet placed on it. The 20 µL reaction mixtures were transferred into vials for UHPLC-HRMS analysis.

### 3.4. LC–MS Analysis

The conditions for performing chromatography were optimized based on previous studies. The LC–MS analysis was conducted using an Agilent high-resolution Q-TOF system series 6520 equipped with an electrospray ionization source (ESI) and UHPLC system series 1290. For the analysis, a Kinetex-C18 (2.1 mm × 50 mm, dp = 1.7 μm) reversed-phase chromatographic column with a C18 precolumn guard (Phenomenex, Torrance, CA, USA) was employed. The MassHunter workstation software version B.06.00 (Agilent Technologies, Santa Clara, CA, USA) was used to control of the UHPLC-HRMS system, manage data acquisition, and perform qualitative and quantitative analysis. To ensure accuracy in mass measurements, a reference mass correction was implemented by utilizing lock masses of 121.050873 and 922.009798 (API-TOF Reference Mass Solution Kit; Agilent Technologies, Santa Clara, CA, USA). The process of optimizing instrument conditions commenced with the tunning of the MS detector in a positive mode operating within an extended dynamic range (2 GHz). Detailed information regarding the chromatographic and spectrometric parameters can be found in Table S1.

### 3.5. Data Preprocessing and Chemometric Studies

Five samples were recorded for optimal time of incubation with HLM and for each investigated electrochemical condition. To eliminate background ion noise in the data and identify the characteristic ions of miconazole degradation products, the molecular feature extraction (MFE) algorithm from Mass Hunter Qualitative Analysis software version B.06.00 (Agilent Technologies, Santa Clara, CA, USA) was employed. After optimizing the parameters of MFE, the following configurations were applied: a maximum permitted charge state of 2 for the analyzed ions, a minimum abundance threshold of 2000 counts for the compound filter, a minimum requirement of 2 ions, and an isotope model tailored for common organic molecules with a peak spacing tolerance 0.0025 *m*/*z*. The multivariate chemometric analysis was conducted using Mass Profiler Professional (MPP) software version 12.61 (Agilent and Strand Life Sciences Pvt. Ltd., Santa Clara, CA, USA) and R software (versions 4.1.0 and 4.3.2, GNU project). All data were centered and scaled prior to chemometric analysis.

### 3.6. In Silico Estimation of Toxicity

Aquatic toxicity, acute toxicity to rodents, mutagenicity, and developmental toxicity of the identified metabolites were assessed using the following software: ECOSAR v. 1.11, ACD Percepta (ACD/Labs 2015 Release, Build 2726), Vega Version 1.1.5-b48 (calculation core version 1.2.8), and Toxicity Estimation Software Tool (T.E.S.T.) version 5.1.1. Receptor-mediated toxicity (endocrine-disruption potential) was calculated with the use of the Endocrine Disruptome platform [26]. In order to visually compare the calculated properties of the metabolites, principal component analysis (PCA) was performed using R 4.1.0 software (GNU Project). All data were centered and scaled prior to chemometric analysis.

## 4. Conclusions

The primary objective of this study was to establish the metabolic pathway of miconazole and elucidate the structures of its metabolites for the first time. During incubation with HLM, the miconazole underwent biotransformation reactions including hydroxylation of the benzene ring and oxidation of the imidazole moiety, along with its subsequent degradation. Notably, seven previously unknown metabolites of miconazole were discovered and identified, which deepens our understanding of its metabolic pathways and transformation processes, shedding light on its environmental fate. This insight aids in the development of improved analytical methods for monitoring miconazole contamination and assessing the environmental impact of miconazole usage based on the newfound knowledge of its metabolites. In silico analysis of toxicity revealed that the metabolites should be generally viewed as less toxic to aquatic species than the parent compound, which aligns with the frequently observed inverse correlation between toxicity and increasing polarity. On the other hand, major metabolites (mainly M1 and M2) may exhibit an elevated risk of mutagenicity and receptor-mediated toxicity, which is particularly important due to the significant risk of endocrine disruptions in non-target organisms. The utilization of multivariate methods enabled the optimization of the electrochemical method using SPE to generate miconazole metabolites. Electrochemical reactions have been proven to be helpful in the realm of metabolic studies, enabling the qualitative simulation of the biotransformation processes of miconazole. The findings of this study demonstrate the capability of electrode processes to mimic oxidation reactions inherent in metabolic pathways. This shows that electrochemical methods can be a compelling supplement to commonly employed metabolic procedures, offering a convenient, easy, quick, and relatively cost-effective means of studying drug metabolism. The UHPLC-HRMS combined system ensured high-resolution MS/MS spectra and allowed for accurate structural characterization of the registered metabolites.

## Figures and Tables

**Table 1:** Accurate mass elemental composition of miconazole and its metabolites.

Name	Retention Time [min]	Mass [*m*/*z*]		Mass Error [ppm]	Molecular Formula [M + H]^+^	Fragmentation MS/MS	
		Measured	Theoretical			Mass [*m*/*z*]	Ion Formula [M + H]^+^
Miconazole	6.01	414.9938	414.9933	1.20	C_18_H_15_Cl_4_N_2_O	379.0206 255.0061 239.0141 227.0137 203.0364 172.9900 158.9764 132.9609 111.0566 69.0457	C_18_H_14_Cl_3_N_2_O C_11_H_9_Cl_2_N_2_O C_11_H_9_Cl_2_N_2_ C_10_H_9_Cl_2_N_2_ C_11_H_8_ClN_2_ C_8_H_7_Cl_2_ C_7_H_5_Cl_2_ C_5_H_3_Cl_2_ C_5_H_7_N_2_O C_3_H_5_N_2_
M1	7.13	448.9887	448.9988	0.22	C_18_H_17_Cl_4_N_2_O_3_	345.9801 273.0218 245.0258 188.0034 170.9801 158.9760 152.0270 117.0601 86.0242	C_14_H_11_Cl_3_NO_3_ C_11_H_11_Cl_2_N_2_O_2_ C_10_H_11_Cl_2_N_2_O C_8_H_8_Cl_2_N C_8_H_5_Cl_2_ C_7_H_5_Cl_2_ C_8_H_7_ClN C_4_H_9_N_2_O_2_ C_3_H_4_NO_2_
M2	7.62	430.9867	430.9882	3.48	C_18_H_15_Cl_4_N_2_O_2_	188.0013 158.9765 85.0408	C_8_H_8_Cl_2_N C_7_H_5_Cl_2_ C_3_H_5_N_2_O
M3	5.27	430.9877	430.9882	1.16	C_18_H_15_Cl_4_N_2_O_2_	174.9709 110.9980 69.0453	C_7_H_5_Cl_2_O C_6_H_4_Cl C_3_H_5_N_2_
M4	5.51	430.9897	430.9882	3.48	C_18_H_15_Cl_4_N_2_O_2_	257.0237 188.9897 174.9708 110.9996 69.0451	C_11_H_11_Cl_2_N_2_O C_8_H_7_Cl_2_O C_7_H_5_Cl_2_O C_6_H_4_Cl C_3_H_5_N_2_
M5	6.47	430.9871	430.9882	2.55	C_18_H_15_Cl_4_N_2_O_2_	243.0078 158.9754 85.0398	C_10_H_9_Cl_2_N_2_O C_7_H_5_Cl_2_ C_3_H_5_N_2_O
M6	5.6	363.9824	363.9824	0	C_15_H_14_Cl_4_NO	188.0025 158.9744 153.0352 117.0563	C_8_H_8_Cl_2_N C_7_H_5_Cl_2_ C_8_H_8_ClN C_8_H_7_N
M7	5.69	421.0018	421.0039	4.99	C_17_H_17_Cl_4_N_2_O_2_	245.02261 188.0033 158.9758	C_10_H_11_Cl_2_N_2_O C_8_H_8_Cl_2_N C_7_H_5_Cl_2_

## Data Availability

Data are contained within the article or Supplementary Materials.

## Supplementary Materials

The following supporting information can be downloaded at: https://www.mdpi.com/article/10.3390/molecules29092160/s1. Section S1. Aquatic toxicity; Table S1. LC-MS parameters; Table S2. List of entities in Principal Component Analysis; Table S3. Toxicity [mg/L] of miconazole and its metabolites to fish (NO SAR—neutral organic SAR, ChV—chronic value, AC_50_—half-maximal activity concentration, NOEC—no observed effect concentration); Table S4. Toxicity [mg/L] of miconazole and its metabolites to daphnia (NO SAR—neutral organic SAR, ChV—chronic value, NOEC—no observed effect concentration); Table S5. Toxicity [mg/L] of miconazole and its metabolites to algae (NO SAR—neutral organic SAR, ChV—chronic value, NOEC—no observed effect concentration); Table S6. Acute toxicity (LD_50_, [log _mg/kg_]) of miconazole and its metabolites to rodents (OR—oral, IP—intraperitoneal, IV—intravenous, SC—subcutaneous); Table S7. Mutagenicity (probability of positive outcome of the Ames test) and developmental toxicity of miconazole and its metabolites; Table S8. Endocrine-disrupting potential of miconazole and its metabolites; colors denote binding probability: green—low, yellow—moderately low, orange—moderately high, red—high (see text for meaning of the numerical values); Figure S1. Overlaid EIC chromatograms of miconazole and its metabolites formed during 120 min incubation with HLM; Figure S2. Cyclic voltammogram of miconazole in formate buffer at pH 3, 7, and 9 (scanning speed of 0.025 V/s); Figure S3. Evolution profiles of major (A) and minor (B) electrochemical reaction products of miconazole; Figure S4. Scree plot showing variance explained by the principal components; Figure S5. Biplot of the first two principal components. Loading numbers correspond to the entity numbers shown in Table S2.
