# Supplementary material for: Identification of New Hepatic Metabolites of Miconazole by Biological and Electrochemical Methods Using Ultra-High-Performance Liquid Chromatography Combined with High-Resolution Mass Spectrometry
Source: Molecules. 2024 May 6;29(9):2160. doi: 10.3390/molecules29092160 (PMC11085085; doi:10.3390/molecules29092160)
Supplement: Supplementary file 1 [file molecules-29-02160-s001.zip › molecules-2953027-supplementary.pdf]

Supplementary material

Identification of new hepatic metabolites of miconazole by biological and electrochemical methods using ultra-high-performance liquid chromatography combined with high-resolution mass spectrometry

Michał Wroński, Jakub Trawiński, Robert Skibiński*

Department of Medicinal Chemistry, Faculty of Pharmacy, Medical University of Lublin, Jaczewskiego 4, 20-090 Lublin, Poland

*Corresponding author. Tel.: +48 81 4487383; fax: +48 81 4487381.

E-mail address: robert.skibinski@umlub.pl

Section S1. Aquatic toxicity

Twenty-three models were employed to calculate toxicity of the miconazole metabolites towards the aquatic organisms, namely six provided by ECOSAR (Neutral Organic SAR: *D. magna* LC_{50} 48 h, Fish LC_{50} 96 h, Green Algae EC_{50} 96 h, *D. magna* Chronic, Fish Chronic and Green Algae Chronic), two provided by T.E.S.T. (Consensus models with the relaxed fragment constraint: *D. magna* LC_{50} 48 h, *F. minnow* LC_{50} 96 h) and fifteen provided by Vega (Zebrafish embryo AC_{50} IRFMN/CORAL (version 1.0.0), Fish Acute LC_{50} Toxicity model KNN/Read-Across (version 1.0.0), Fish Acute LC_{50} Toxicity model NIC (version 1.0.0), Fish Acute LC_{50} Toxicity model IRFMN (version 1.0.0), Fish Acute LC_{50} Toxicity model IRFMN/Combase (version 1.0.0), Fish Chronic NOEC Toxicity model IRFMN (version 1.0.0), *F. minnow* LC_{50} 96h EPA (version 1.0.7), *D. magna* LC_{50} 48h EPA (version 1.0.7), *D. magna* LC_{50} 48h DEMETRA (version 1.0.4), *D. magna* Acute EC_{50} Toxicity model IRFMN (version 1.0.0), *D. magna* Acute EC_{50} Toxicity model IRFMN/Combase (version 1.0.0), *D. magna* Chronic NOEC Toxicity model IRFMN (version 1.0.0), Algae Acute EC_{50} Toxicity model IRFMN (version 1.0.0), Algae Acute EC_{50} Toxicity model ProtoQSAR/Combase (version 1.0.0), Algae Chronic NOEC Toxicity model IRFMN (version 1.0.0)).

Table S1. LC-MS parameters

Device	Parameter	Value
LC	Solvents	A – acetonitrile
		B – 0.1% aqueous solution of HCOOH
	Gradient	5% A to 80% A (0-9 min)
	Analysis time	9 min
	Post-time equilibration	2.50 min (5%A solvent)
	Flow rate	0.3 mL min^{-1}
	Injection volume	2 µL
	Column temperature	35 °C
MS	Ion source	Electrospray (ESI)
	Mode	Positive
	Source temperature	300 °C
	Drying gas flow	10 L min^{-1}
	Nebulizer pressure	40 psig
	Capillary voltage	3500 V
	Fragmentor voltage	140 V
	Skimmer voltage	65 V
	Octopole voltage	750 V
	Mass range	90 - 950 m/z
	Acquisition rate	1.5 spectra s^{-1}

Table S2. List of entities in Principal Component Analysis

No.	m/z	Retention time [min]
1	259.0214	2.88
2	273.0202	7.07
3	275.0161	7.07
4	363.9822	5.59
5	365.9795	5.59
6	367.9762	5.59
7	397.0262	5.16
8	421.0034	5.66
9	423.0007	5.66
10	430.9874	5.15
11	430.9877	5.50
12	430.9876	6.40
13	430.9876	7.60
14	430.9876	5.25
15	432.9849	6.40
16	432.9851	7.59
17	449.0024	7.07

Table S3. Toxicity [mg/L] of miconazole and its metabolites to fish (NO SAR – neutral organic SAR, ChV – chronic value, AC_{50} – half-maximal activity concentration, NOEC – no-observed effect concentration)

Cpd.	Fish LC_{50} 96h NO SAR	Fish ChV	F. minnow LC_{50} 96h T.E.S.T.	Zebrafish embryo AC_{50} IRFMN/CORAL 1.0.0	Fish Acute LC_{50} KNN/Read-Across 1.0.0	Fish Acute LC_{50} NIC 1.0.0	Fish Acute LC_{50} IRFMN 1.0.0	Fish Acute LC_{50} IRFMN/Combase 1.0.0	Fish Chronic NOEC IRFMN 1.0.0	F. minnow LC_{50} 96h EPA 1.0.7
Mic	0.052	0.008	0.13	0.54094	0.3016	1.51	0.677	1.1	0.0048	0.2162
M1	14.013	1.627	0.2	0.32842	0.2954	1.63	0.7749	1.19	0.0173	5.52
M2	0.521	0.072	0.13	0.64172	0.2961	1.56	1.01	1.31	0.0125	0.7076
M3	0.146	0.021	0.11	1.54961	0.5593	1.56	0.7414	1.6	0.004	0.2857
M4	0.146	0.021	0.1	3.38494	0.5445	1.56	0.9062	2.23	0.0037	0.2645
M5	0.031	0.005	0.0846	0.10337	0.2516	1.56	0.8168	1.51	0.0043	0.1397
M6	0.597	0.081	0.33	0.31354	0.3147	1.64	0.7946	1.77	0.0155	0.4619
M7	1.134	0.15	0.84	0.35222	0.2994	1.53	0.7492	0.1572	0.0137	4.06

Table S4. Toxicity [mg/L] of miconazole and its metabolites to daphnia (NO SAR – neutral organic SAR, ChV – chronic value, NOEC – no-observed effect concentration)

Cpd.	D. magna LC_{50} 48h NO SAR	D. magna ChV	D. magna 48h T.E.S.T.	D. magna LC_{50} 48h EPA 1.0.7	D. magna LC_{50} 48h DEMETRA 1.0.4	D. magna Acute EC_{50} IRFMN 1.0.0	D. magna Acute EC_{50} Toxicity model IRFMN/Combase	D. magna Chronic NOEC IRFMN 1.0.0
Mic	0.044	0.013	0.35	1.76	0.1819	0.0543	1.13	0.0976
M1	9.209	1.349	0.97	0.1756	0.445	0.4097	0.0646	0.5542
M2	0.396	0.087	0.38	7.74	0.1999	0.3807	0.1188	0.0937
M3	0.117	0.03	0.31	3.72	2.81	0.1455	0.0042	0.1957
M4	0.117	0.03	0.35	3.31	2.95	0.0955	0.0008	0.2335
M5	0.027	0.008	0.27	3.22	0.2878	0.0439	0.2704	0.3477
M6	0.448	0.095	0.11	0.1817	0.2484	0.3052	0.0062	0.2602
M7	0.831	0.165	0.11	0.0584	0.5314	0.2503	0.0447	0.2753

Table S5. Toxicity [mg/L] of miconazole and its metabolites to algae (NO SAR – neutral organic SAR, ChV – chronic value, NOEC – no-observed effect concentration)

Cpd.	GA 96h (EC_{50}) NO SAR	GA ChV	Algae Acute EC_{50} IRFMN 1.0.0	Algae Acute EC_{50} ProtoQSAR/Combase	Algae Chronic NOEC IRFMN 1.0.0
Mic	0.165	0.104	0.4407	0.2429	0.1364
M1	12.552	4.551	0.197	0.1023	0.2297
M2	0.983	0.493	0.1768	0.2238	0.1684
M3	0.368	0.209	0.4804	0.017	0.1801
M4	0.368	0.209	0.452	0.0523	0.1888
M5	0.111	0.074	0.3881	0.1087	0.1793
M6	1.051	0.511	0.1068	0.3621	0.0931
M7	1.781	0.824	0.1681	0.1324	0.1782

Table S6. Acute toxicity (LD_{50}, [log $_{mg/kg}$]) of miconazole and its metabolites to rodents (OR – oral, IP – intraperitoneal, IV – intravenous, SC – subcutaneous)

Cpd.	Rat OR T.E.S.T.	Mouse IP	Mouse OR	Mouse IV	Mouse SC	Rat IP	Rat OR
Mic	2.41	2.39	2.97	1.78	2.74	2.68	3.14
M1	2.48	2.65	3.19	2.04	2.27	3.03	3.4
M2	2.05	2.83	3.06	2.28	2.76	2.77	3.08
M3	2.33	2.33	3.15	1.93	2.54	2.67	2.7
M4	2.33	2.4	3.14	1.75	1.7	2.61	3.16
M5	2.56	2.51	3.11	1.86	2.77	2.75	3.24
M6	3.37	2.46	2.84	1.81	1.88	2.53	2.79
M7	2.33	2.68	2.98	1.92	2.3	2.62	3.31

Table S7. Mutagenicity (probability of positive outcome of the Ames test) and developmental toxicity of miconazole and its metabolites

Cpd	Mutagenicity T.E.S.T.	Mutagenicity Percepta	Developmental toxicity
Mic	0.5	0.07	0.43
M1	-0.02	0.11	1.06
M2	0.82	0.14	0.71
M3	0.17	0.06	0.66
M4	0.18	0.07	0.66
M5	0.01	0.07	0.78
M6	0.27	0.06	0.56
M7	0.23	0.09	0.99

Table S8. Endocrine disrupting potential of miconazole and its metabolites; colors denote binding probability: green – low, yellow – moderate low, orange – moderately high, red – high (see text for meaning of the numerical values)

	AR	AR an[a]	ERα	ERα an	ERβ	ERβ an	GR	GR an	LXRα	MR	PPARγ	PR	RXRα	TRα	TRβ
Mic	-5.8	-6.7	-8.3	-8.4	-7.3	-8.2	-8.7	-7.7	-9.8	-9.0	-8.6	-2.6	-9.3	-7.2	-7.9
M1	-5.7	-7.4	-8.1	-8.7	-6.6	-9.2	-9.0	-8.5	-9.7	-9.1	-8.6	-2.6	-9.8	-5.8	-8.3
M2	-5.3	-6.5	-8.4	-8.5	-7.0	-8.3	-8.6	-8.0	-9.8	-9.2	-8.9	-2.6	-10.1	-7.4	-8.6
M3	-4.9	-6.7	-8.6	-8.1	-7.3	-8.3	-8.6	-8.0	-9.5	-8.8	-8.6	-2.7	-9.4	-6.9	-8.0
M4	-4.4	-6.2	-8.0	-8.2	-5.7	-7.7	-8.5	-8.1	-9.6	-8.3	-7.6	-2.6	-9.5	-6.8	-7.5
M5	-5.7	-7.3	-8.1	-8.2	-6.3	-8.4	-8.6	-8.2	-9.9	-9.1	-8.8	-2.9	-9.8	-7.4	-8.7
M6	-6.3	-6.9	-8.1	-7.7	-7.0	-8.0	-8.2	-7.3	-9.3	-7.8	-8.1	-2.3	-8.3	-7.3	-8.6
M7	-6.1	-7.7	-8.1	-8.5	-6.7	-8.4	-9.0	-7.6	-10.1	-9.0	-9.3	-2.4	-9.6	-7.0	-8.7

[a] – antagonistic

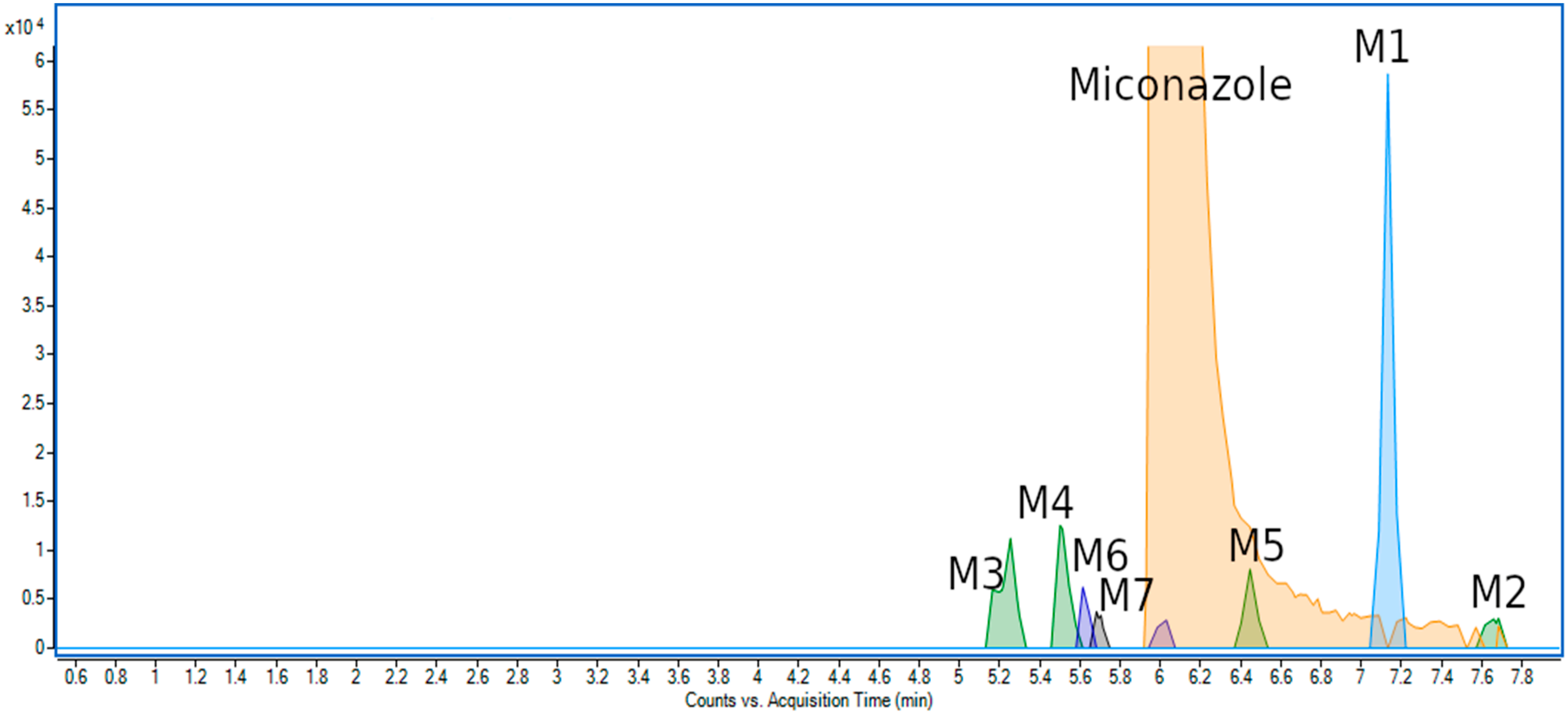

Figure S1. Overlaid EIC chromatograms of miconazole and its metabolites formed during 120 min incubation with HLM.

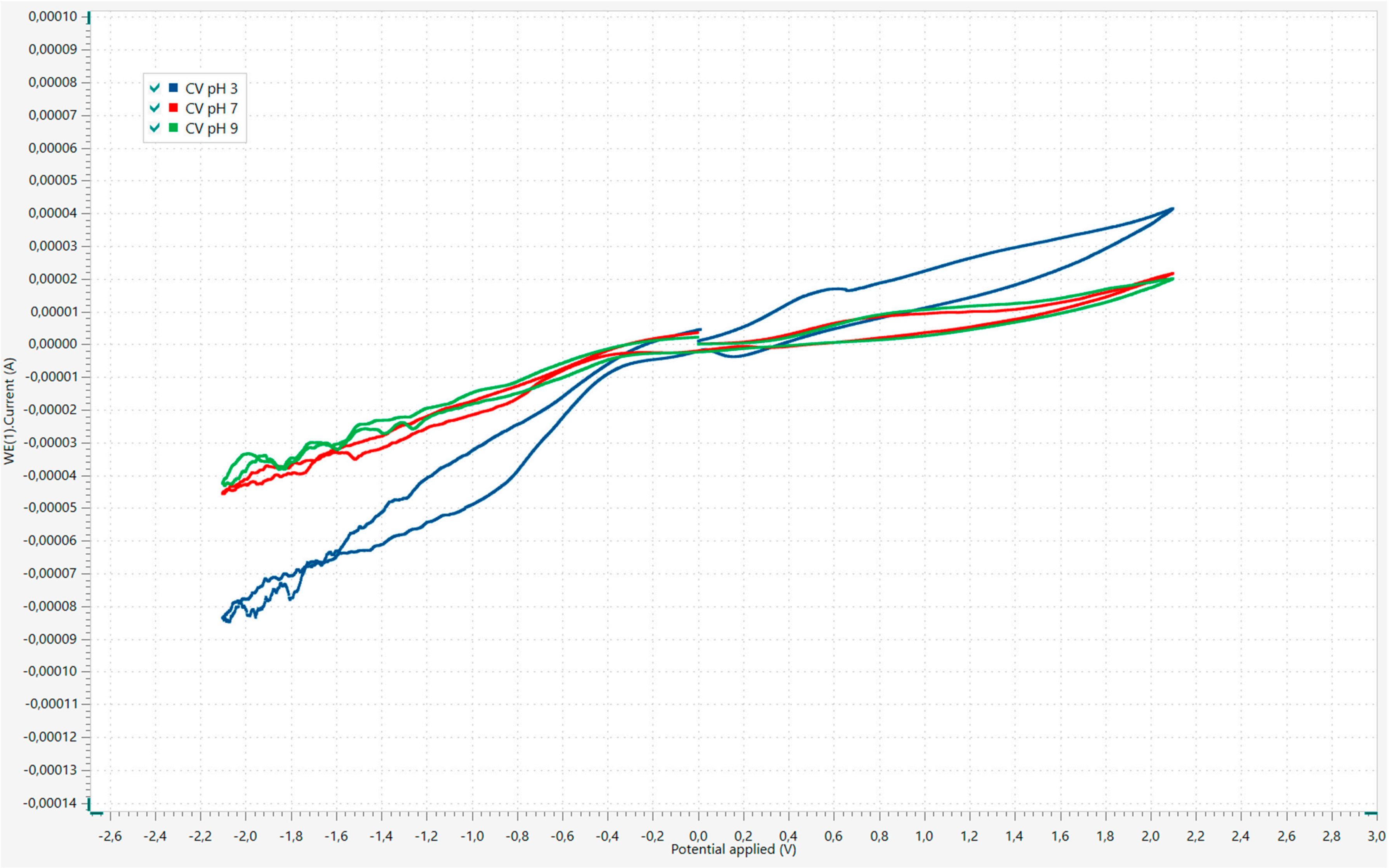

Figure S2. Cyclic voltammogram of miconazole in formate buffer at pH 3, 7, and 9 (scanning speed of 0.025 V/s).

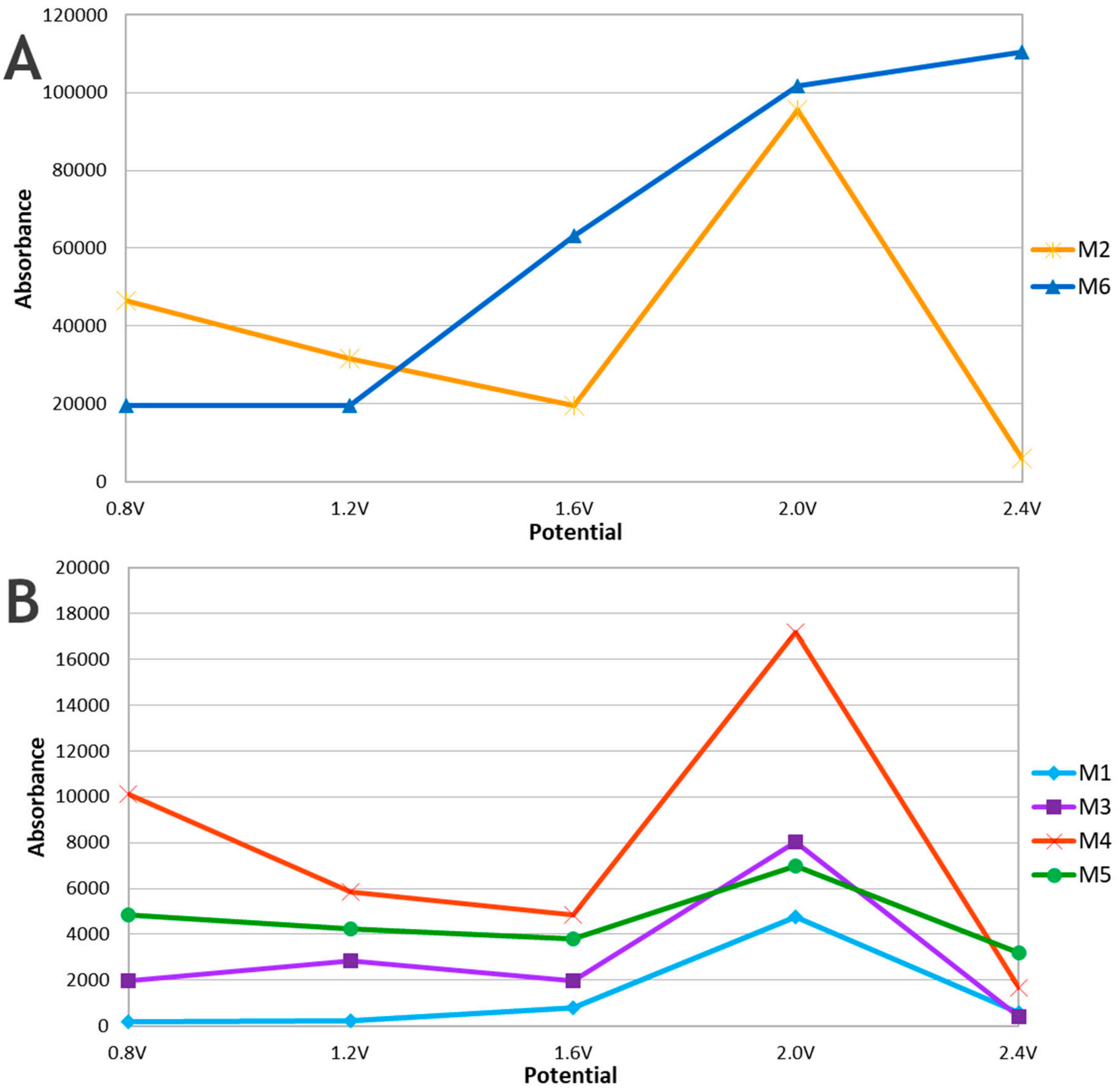

Figure S3. Evolution profiles of major (A) and minor (B) electrochemical reaction products of miconazole.

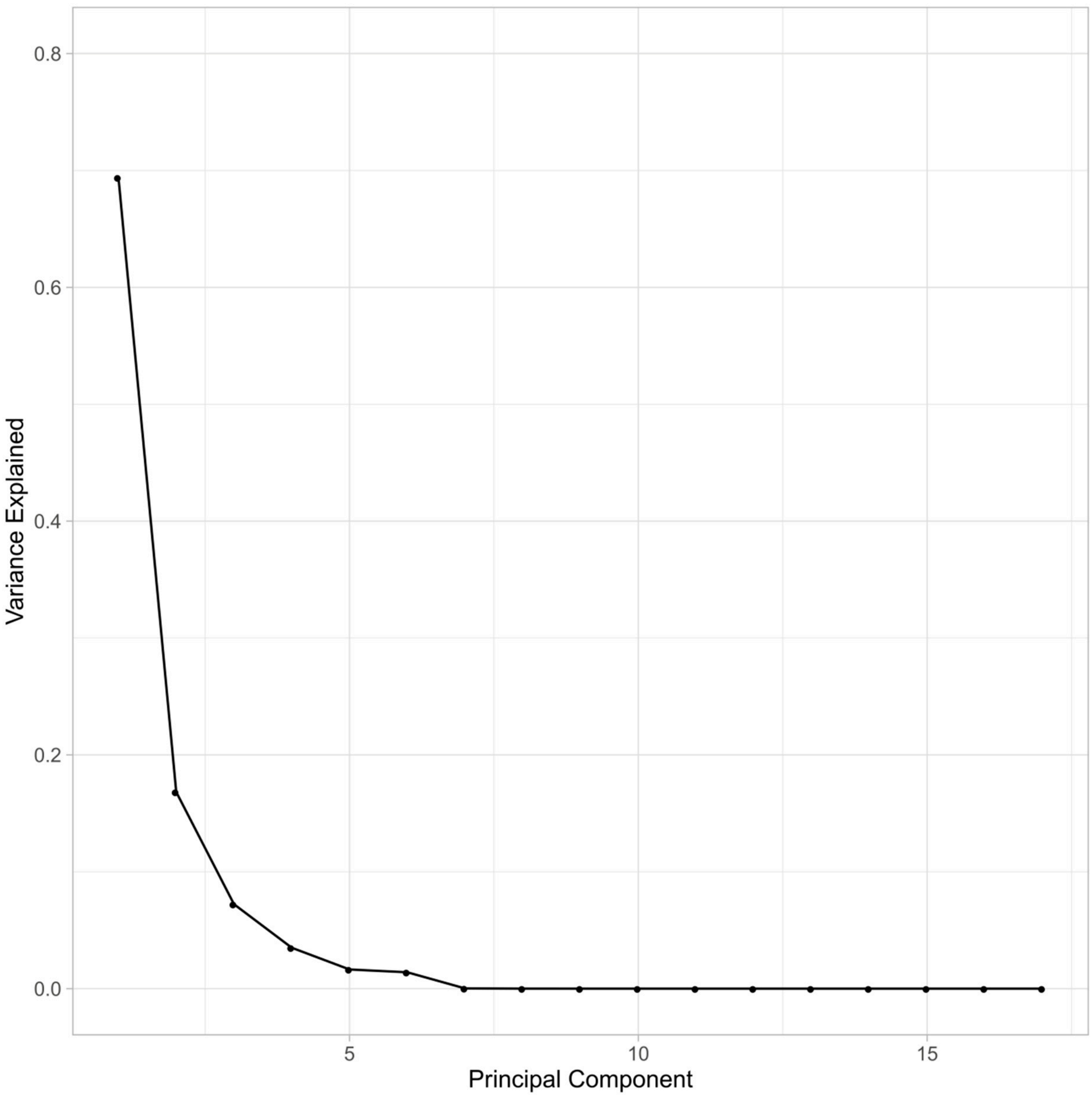

Figure S4. Scree plot showing variance explained by the principal components.

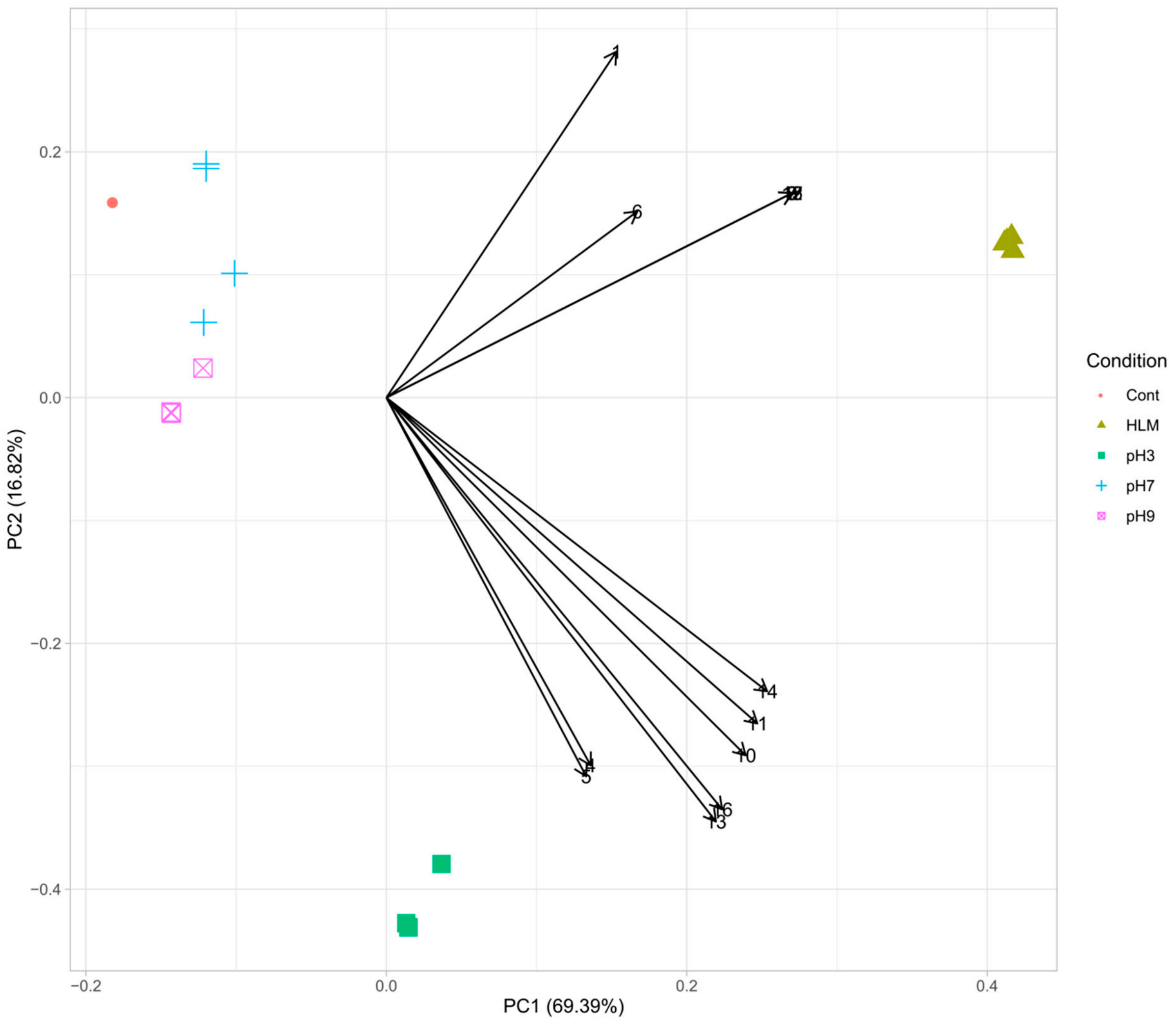

Figure S5. Biplot of the fisrt two principal components. Loadings numbers correspond to the entities numbers shown in Table S2.
